# Appropriate sampling to aid on‐farm assessments of the haplotype composition of *Zymoseptoria tritici* populations

**DOI:** 10.1002/ps.8454

**Published:** 2024-10-11

**Authors:** Catherine Harrison, Neil Boonham, Roy Macarthur, Michael David Parr, Femke van den Berg

**Affiliations:** ^1^ Plants Program Fera Science Ltd., York Biotech Campus York UK; ^2^ School of Natural and Environmental Sciences Newcastle University Newcastle upon Tyne UK

**Keywords:** fungicide resistance, *Zymoseptoria tritici*, haplotype, CYP51, azole

## Abstract

**BACKGROUND:**

*Zymoseptoria tritici* causes Septoria tritici blotch (STB), which is the biggest threat to wheat in the UK. Azole fungicides have been used since the 1980s to control STB, but resistance to these chemicals is now widespread. The main resistance mechanism is based on the accumulation of CYP51 mutations, with 33 mutations reported. Hence, farmers need an accurate estimate of the haplotype composition of *Z. tritici* populations to develop effective fungicide treatments and resistance management.

**RESULTS:**

Isolates from *Z. tritici* lesions were collected from three fields across three commercial farms using two sampling approaches. Analysis of the isolate sequences revealed that the number of distinct haplotypes and the haplotype composition of the most dominant haplotypes varied only between and not within farms. Conventional W‐shaped and point sampling both found the same percentage of distinct haplotypes and frequencies of the six most dominant haplotypes.

**CONCLUSION:**

The results from this survey suggest that farm‐resistance‐management strategies should be based on farm‐specific rather than national data, and that sampling within a single field is sufficient. W‐shaped sampling is often recommended in sampling approaches, but this survey finds no evidence of this approach being more appropriate for detecting a greater percentage of distinct haplotypes which may aid the discovery of potential new resistance threats. © 2024 Fera Science Ltd. *Pest Management Science* published by John Wiley & Sons Ltd on behalf of Society of Chemical Industry.

## Graphical abstract

Sanger sequencing of *Zymoseptoria tritici* lesions on wheat plants were used to derive a minimum spanning haplotype network.

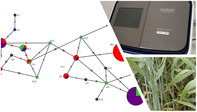

## 1 INTRODUCTION

*Zymoseptoria tritici* (also known by the synonym *Mycosphaerella graminicola*) is an ascomycete fungus which causes Septoria tritici blotch (STB). It is distributed globally and is a significant threat to wheat production, especially in areas with high rainfall during the growing season.^1^ It is the most common foliar disease of wheat in Europe and the biggest threat to wheat in the UK. In the UK, foliar diseases of wheat account for losses of ≤20% in attainable yield.^2^ The average annual cost of yield losses are estimated at £53 M, with a maximum cost of £173 M based on a wheat price of £125 t^−1^. Most fungicides used on wheat are targeted against STB control and when the costs of these fungicides are included, these annual costs rise to £112 M and £253 M, respectively.^3^

Primary infection of a crop occurs through airborne ascospores (sexual) released from pseudothecia present in crop debris. Secondary spread is driven by rainwater and rain splash of pycnidiospores (asexual) causing local spread to nearby leaves.^4^ The pycnidia are produced 14 to 40 days postinfection, depending on the environmental conditions. Many cycles of both sexual and asexual reproduction occur during the growing season, allowing the disease to spread rapidly.^5^ STB disease is characterised by leaf cell collapse leading to irregular necrotic lesions containing fruiting bodies. Lesions reduce the green leaf area of the plant preventing photosynthesis and, when present on upper leaves during grain filling, result in a reduced yield.^6^

Fungicides from different classes of compounds have been used for disease control of STB. In the 1970s benzimideazole fungicides were introduced which have both curative and long‐protective activity, and were used exclusively until the evolution of resistant strains rendered them ineffective.^7^ From the 1980s onwards, sterol demethylation inhibitors (DMIs) such as azole fungicides have become extensively used. More recently, the broad‐spectrum systemic strobilurins (QoIs) fungicides and succinate dehydrogenase inhibitor (SDHI) fungicides have been developed and introduced for the control of *Z. tritici*.

*Zymoseptoria tritici* has rapidly developed resistance to strobilurins as a result of continued selective pressure from prophylactic use with resistance being detected for the first time in 2000, reaching 70% prevalence in populations by 2003.^8^ By contrast, resistance to azoles has been much slower to develop,^9^ despite having been in use for over 30 years. Azoles target CYP51, cytochrome P450 sterol 14 α‐demethylase (CYP51), an enzyme involved in the production of sterols, which is important for the regulation of cell membrane permeability.

Both target site and nontarget site fungicide resistance mechanisms have been identified in plant pathogens.^10^ Mutations causing amino acid alterations in the CYP51 gene result in a decreased affinity of the protein for specific compounds and lead to target site resistance. Overexpression of the target CYP51 gene, which is most frequently caused by insertions in the predicted promoter regions and overexpression of genes encoding efflux pumps result in nontarget site resistance to a range of compounds.^10^ These mechanisms can combine, which means that resistance levels in fungal strains are often determined by combinations of CYP51 amino acid alterations, CYP51 gene overexpression and/or enhanced efflux.

To date, 33 mutations have been reported with increasing incidence of mutations being found in combination.^11^, ^12^ Huf *et al*.^12^ published a ‘new' nomenclature to classify the different combinations of mutations in the *CYP51* gene, with a letter denoting the number of amino acid alterations in the CYP51 enzyme and a number referring to the specific combination of mutations. Over 100 different mutation combinations (haplotypes) have been identified, although it is believed that nine main haplotypes represent 85% of the European population.^12^

As part of a recent European survey of DMI and SDHI fungicide resistance of *Z. tritici* Hellin, Duvivier *et al*.^13^ developed a standardised leaf sampling protocol using the S524T mutation as an indicator of azole sensitivity across the wider European *Z. tritici* population. The S524T mutation has been shown to be correlated with a significant increase in resistance irrespective of the other mutations present within the isolate and hence this method provides an excellent cost‐effective first assessment of the potential yield loss risks associated with an increase in azole resistance on a large geographical scale. However, local farm‐scale *Z. tritici* populations could consist mainly of haplotypes that do not contain the S524T mutation but still contain significant frequencies of haplotypes associated with a reduced azole sensitivity. Additionally, there may be haplotypes containing novel mutations associated with an increased resistance which may ultimately become more dominant and more widely spread if they have a competitive advantage. Moreover, a recent study by Ballu *et al*.^14^ suggests that the population haplotype composition is the principal factor to consider when designing sustainable field‐based resistance management strategies. Effective on‐farm resistance management would hence require a survey approach that detects the haplotype population composition.

Brunner *et al*.^9^ suggest that CYP51 mutations generally only arise once locally and are subsequently spread through wind‐dispersed ascospores. They suggest that subsequent selection of novel less‐sensitive haplotypes is the result of recurring cycles of recombination and selection resulting from widespread azole use. Such rapid local adaptation could result in initial clustering of newly arisen haplotypes which may ultimately become more dominant and more widely spread if they have a competitive advantage. If such haplotype clustering occurs, the sampling locations and methods used within the survey may affect how well the haplotype population composition experienced under field conditions will be reflected within the survey samples. More specifically, clustering may affect, amongst other things, whether a farm resistance management strategy can be based on national average or regional resistance data or whether it needs to be farm‐ or field‐specific.

The aim of this study was hence to determine an appropriate Z. *tritici* resistance screening strategy to (ultimately) aid on‐farm resistance management.

## 2 MATERIALS AND METHODS

### 2.1 Sampling protocol

Wheat plants were collected in April 2015 before fungicide treatment. Samples were collected from three fields on three UK commercial farms in Dorset, Salisbury (Wiltshire) and Louth (Lincolnshire), where STB control had failed the previous year. At the time of sample collection, the crop was approaching growth Stage 30 (stem elongation). Two different methods of sampling were employed in this study: normal sampling (referred to with ‘W’) and point sampling (referred to with ‘H’). For the W sampling, three plants were collected from 20 positions in a W‐grid pattern (60 plants per field) ensuring samples were collected evenly across the field. For H sampling, 60 plants were collected from a single location. To ensure that there was no bias in the results, samples were tested in a randomised order.

### 2.2 Isolation of *Zymoseptoria tritici*

Isolation of *Z. tritici* from leaf samples was carried out using a protocol modified from Dooley.^15^ In summary, three symptomatic leaves were taken from each plant. Where no symptomatic leaves were present, one inner leaf and two outer leaves were tested (typically leaves 4, 2 and 1). Leaves were rehydrated by soaking them in sterile distilled water for a minimum of 1 min before being surface‐sterilised by immersion in 10% sodium hypochlorite for 30 s and rinsed in sterile distilled water). Leaves were dried using filter paper (Whatman) before being placed onto tap water agar (TWA) with the pycnidia facing upwards. Plates were incubated for 48 h at 20 °C with a UV light source. After incubation, for leaves showing symptoms, a single cirrus per leaf was isolated using a sterile needle before being streaked onto potato dextrose agar (PDA) amended with 1% streptomycin. Plates were incubated for 5–7 days at 20 °C with a UV light source. Isolates were subcultured onto fresh PDA plates amended with 1% streptomycin until pure cultures were attained. Pure cultures were transferred to 10% sterile skimmed milk for storage at −30 °C.

### 2.3 *CYP51* DNA analysis

DNA was extracted using the QIAamp DNA mini kit (Qiagen GmbH, Hilden, Germany) following the manufacturer's protocol and then stored at −30 °C until required. The *CYP51* gene was amplified using PCR with one unit of Phusion® hot start flex DNA polymerase (New England Biolabs, Ipswich, MA, USA), 200 μm each dNTP, 0.5 μm each primer, 3% DMSO and 1 × Phusion® HF buffer in a final volume of 50 μL. Primers were adapted from Fraaije *et al*.^16^ (ST51F1–5′ ATGGGTCTCCTCCAGGAACTCCTCC 3′, ST51R3–5′ TTGTGAAAGCAGCGTCTCCCTC 3′). The following cycling conditions were used: 98 °C for 30 s followed by 35 cycles of 98 °C for 10 s, 68 °C for 20 s, and 72 °C for 1 min and a final extension step of 72 °C for 10 min. PCR products were separated on 1.2% agarose gels in TBE (90 mm Tris/borate/EDTA) buffer containing ethidium bromide and visualised under UV light.

Amplified DNA was Sanger‐sequenced using primers modified from Fraaije *et al*.,^16^ (i.e. SF ‐5′ GCGCAGTTCGACGCGCAATT 3′, F3‐5′ GCGGACCTCTACCACTACCTCGA 3′, SR2‐5′CGCGCTATTCATTAGCATAACATCCACC 3′; Eurofins Genomics, Ebersberg, Germany). Sequences were assembled and trimmed using sequencer scanner 2 (Applied Biosystems, Foster City, CA, USA), and aligned and analysed for the presence of single nucleotide polymorphisms (SNPs) using Mega version 6.^17^ The upstream regulation region of the CYP51 gene was sequenced as described above using primers Mg51‐proF‐5′ GTGGCGAGGGCTTGACTAC 3′ and Mg51‐seqR‐5′ CTGCGCGAGGACTTCCTGGA 3′^18^ for both amplification and sequencing.

### 2.4 Statistical analysis

Statistics and data manipulations were performed within R (v4.1.2^19^) relying on the in‐built stats package.

#### 2.4.1 Frequencies of most common haplotypes

Differences between samples were calculated through the vegan package,^20^ utilising an unweighted Jaccard distance to quantify differences in the presence of haplotypes, and an abundance weighted Jaccard distance, also referred to as a Ruzicka distance, to quantify differences in the presence and relative proportion of haplotypes. To test differences between factors, a permutational ANOVA (*n* = 10 000) was employed to quantify the effect of farm, field and sampling method. Significant results were then interrogated using PCA. This analysis focused on the number of isolates attributed to the haplotypes that contribute to >80% of all isolates across the full survey, and thereby assesses whether there is evidence for interfield and interfarm variability in haplotype frequencies of the most dominant haplotypes, and whether the frequencies identified for these haplotypes are significantly affected by the sampling method.

#### 2.4.2 Percentage of distinct haplotypes

Analysis of variance (ANOVA) tests were used to quantify the variability in the number of distinct haplotypes found (No. distinct) and the percentage of distinct haplotypes found within the isolates tested (P. distinct) between farm, field and sampling method. Significant values were interrogated using a paired Tukey's honestly significant difference (HSD) test or a Wilcoxon signed rank test with continuity correction for No. distinct and P. distinct, respectively. This analysis assesses the between‐field and ‐farm variability in the ability to detect more rare haplotypes, and whether this is affected by the sampling method used.

## 3 RESULTS

### 3.1 Haplotype recovery from leaf samples

At each commercial farm, leaves were collected from three fields using two sampling methods (H & W) which led to the collection of 360 leaves per farm, giving 1080 leaves in total. From these leaves, a total of 250 cultures were established and of these 229 were sequenced with sufficiently high quality for analysis.

### 3.2 Summary of haplotypes found within the surveys

**Figure 1:**
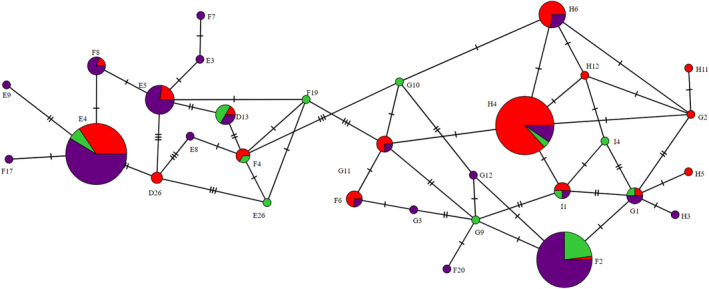
Minimum spanning haplotype network for Z*ymoseptoria tritici* representing the haplotype distribution between farms (green = Louth; purple = Salisbury; red = Dorset) amongst all isolates that were successfully sequenced. Each circle depicts a unique haplotype and the size of each circle is proportional to the number of isolates of that haplotype. CYP51 amino acid mutations are denoted by the hatch marks across lines connecting haplotypes with each hatch mark representing a mutation.

The sequence data resulted in the identification of between 12 to 17 distinct haplotypes at each of the three farms: 17 distinct haplotypes from 97 isolates collected at the Dorset farm, 20 distinct haplotypes from 108 isolates collected at the Salisbury farm, and 12 distinct haplotypes from 28 isolates collected at the Louth farm (Table 1). However, across all three farms only a total of 31 distinct haplotypes were detected, suggesting significant overlap in haplotypes between farms. At Salisbury, there were 12 occurrences in which a single isolate of a particular haplotype was found, whereas this occurred eight times at the Louth farm and the Dorset farm (Table 1). Haplotype H4 was most prevalent at the Dorset farm, whereas F2 was most prevalent at the Salisbury and Louth farms (Fig. 1).

**Table 1:** Overview of the number of the number and percentage of distinct haplotypes found within commercial crops at three farms in the UK during April 2015. Results are presented for samples taken across three fields per farm using two different sampling methods; point (‘H’) sampling (60 samples from a single position within the field) and conventional W‐shaped ‘W’ sampling (three samples taken at 20 positions across the W; 60 leaves in total). No. distinct, number of distinct haplotypes found; P. distinct, percentage of distinct haplotypes found within the isolates tested [(No. distinct/Number of isolates sequenced) × 100]; and No. one, number of occurrences of finding a single isolate

Farm	Field	Sampling method	Number of isolates sequenced	No. distinct	P. distinct (%)	No. one
Dorset	F1	H	20	5	25	2
		W	21	8	38	5
	F1 total		41	9	22	4
	F2	H	22	7	32	3
		W	10	6	60	4
	F2 total		32	10	31	6
	F3	H	11	5	45	2
		W	13	3	23	1
	F3 total		24	5	21	1
	Farm total		97	17	18	8
Salisbury	F1	H	25	10	40	8
		W	18	6	33	2
	F1 total		43	12	28	6
	F2	H	23	7	30	3
		W	13	5	38	2
	F2 total		36	9	25	4
	F3	H	9	4	44	2
		W	20	7	35	4
	F3 total		29	9	31	6
	Farm total		108	20	19	12
Louth	F1	H	3	2	67	1
		W	4	4	100	4
	F1 total		7	4	57	2
	F2	H	10	3	30	1
		W	5	5	100	5
	F2 total		15	7	47	5
	F3	H	3	3	100	3
		W	3	2	67	1
	F3 total		6	5	83	4
	Farm total		28	12	43	8

The mutation S524T, which is common across Europe, and which is associated with a significantly reduced azole sensitivity was present in only 49% of all isolates screened in this survey. More specifically, at the individual farms the mutation was detected in only 28% of isolates at Salisbury and 39% at Louth. The mutation was, however, much more frequent at the Dorset farm and was detected in 74% of the isolates (see Supporting Information, Tables S1 and S2). Isolates allocated to haplotypes E4 and F2, which made up >45% of the isolates within the survey, are among the 12 haplotypes found that do not contain the S524T mutation.

### 3.3 Within‐ and between‐farm haplotype variability

#### 3.3.1 Frequencies of six overall most common haplotypes

**Figure 2:**
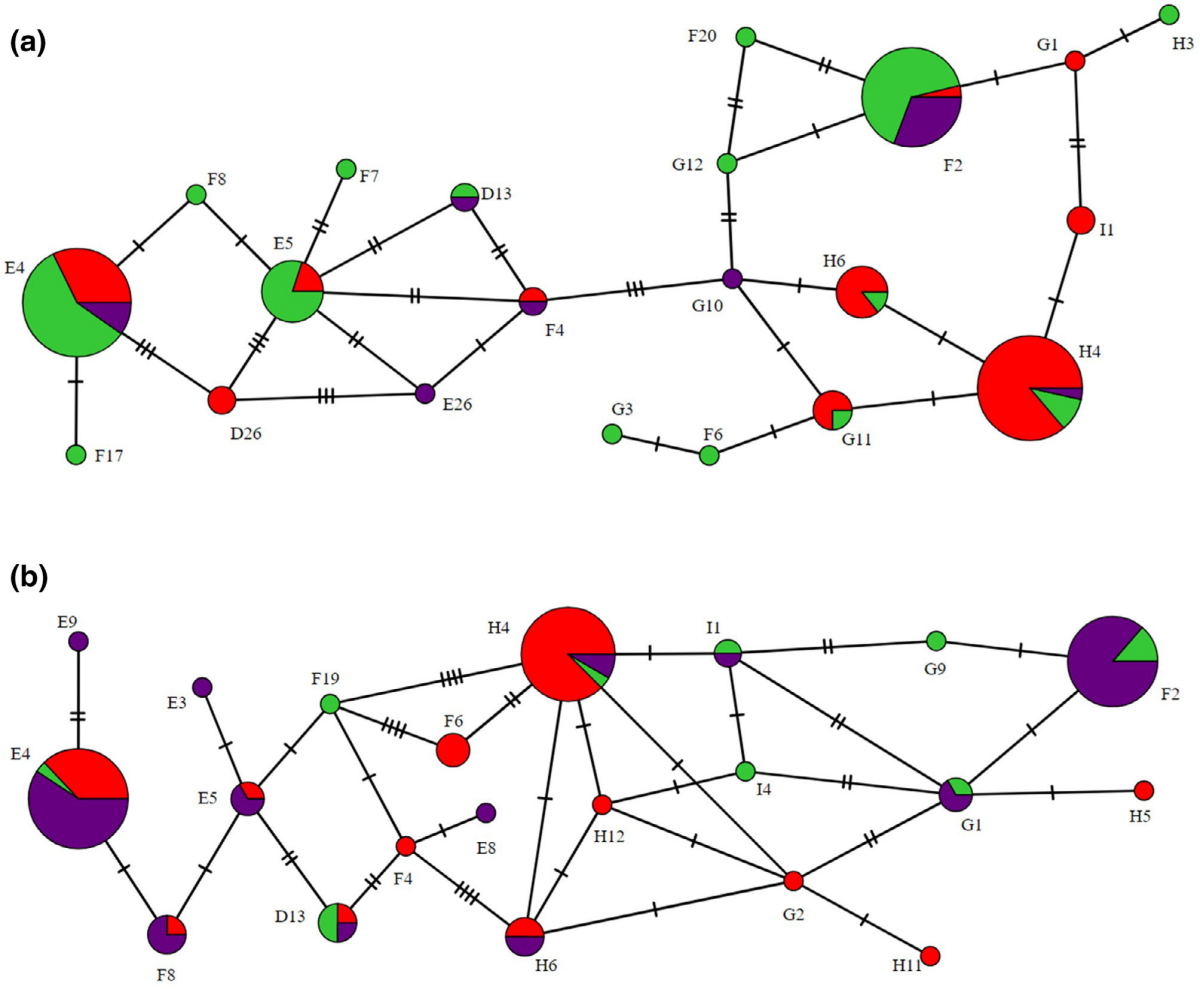
Minimum spanning haplotype network for Z*ymoseptoria tritici* representing the haplotype distribution between farms (green = Louth; purple = Salisbury; red = Dorset) amongst all isolates that were successfully sequenced after collection using (a) the H sampling method (point sampling at two locations within a field) and (b) the W sampling method (conventional W‐shaped sampling). Each circle depicts a unique haplotype and the size of each circle is proportional to the number of isolates of that haplotype. CYP51 amino acid mutations are denoted by the hatch marks across lines connecting haplotypes with each hatch mark representing a mutation.

The six most common haplotypes of *Z. tritici* across the full survey were D13, E4, E5, F2, H4 and H6 which made up 81% of samples (Fig. 1). When considering only isolates collected through H sampling this was very similar, although G11 was more frequent than D13 [Fig. 2(a)]. When only considering isolates collected through W sampling, D13, E4, F2, H4 and H6 remain amongst the six most common haplotypes, but E5 does not [Fig. 2(b)]. Instead, F8 and H6 are present at a frequency equal to that of D13 which represents the lowest frequency of the most common isolates (Table S1). However, the haplotypes that were most common across the whole survey represented similar total frequencies for both methods, with these six haplotypes representing 81.8% of all isolates collected with the H sampling method and 74.8% of isolates collected with the W sampling method (Tables 2 and 3).

**Table 2:** Overview of the percentage of all isolates sequenced (*n* = 107) from samples collected using the W sampling that belong to one of the six overall most common haplotypes and the total percentage isolates found by W sampling that belong to these haplotypes

Farm	Field	D13	E4	E5	F2	H4	H6	Total
Dorset	F1	0.00	2.80	0.93	0.00	9.35	0.93	14.02
	F2	0.93	1.87	0.00	0.00	3.74	0.93	6.54
	F3	0.00	4.67	0.00	0.00	6.54	0.00	11.21
Farm total		0.93	9.35	0.93	0.00	19.63	1.87	31.78
Salisbury	F1	0.00	3.74	0.93	7.48	1.87	0.00	14.02
	F2	0.00	2.80	0.93	5.61	0.00	1.87	11.21
	F3	0.93	8.41	0.00	4.67	0.00	0.00	13.08
Farm total		0.93	14.95	1.87	17.76	1.87	1.87	38.32
Louth	F1	0.93	0.00	0.00	0.93	0.00	0.00	0.93
	F2	0.93	0.93	0.00	0.00	0.00	0.00	0.93
	F3	0.00	0.00	0.00	1.87	0.93	0.00	2.80
Farm total		1.87	0.93	0.00	2.80	0.93	0.00	4.67
Overall total		3.74	25.23	2.80	20.56	22.43	3.74	74.77

**Table 3:** Overview of the percentage of all isolates sequenced (*n* = 126) from samples collected using the H sampling that belong to one of the six overall most common haplotypes and the total percentage isolates found by H sampling that belong to these haplotypes

Farm	Field	D13	E4	E5	F2	H4	H6	Total
Dorset	F1	0.00	2.38	0.79	0.00	10.32	1.59	15.08
	F2	0.00	3.17	0.79	0.79	6.35	3.17	14.29
	F3	0.00	2.38	0.00	0.00	3.17	0.00	5.56
Farm total		0.00	7.94	1.59	0.79	19.84	4.76	34.92
Salisbury	F1	0.79	7.14	0.79	6.35	0.00	0.79	15.08
	F2	0.00	3.97	4.76	4.76	2.38	0.00	15.87
	F3	0.00	3.17	0.79	2.38	0.00	0.00	6.35
Farm total		0.79	14.29	6.35	13.49	2.38	0.79	37.30
Louth	F1	0.79	0.00	0.00	1.59	0.00	0.00	1.59
	F2	0.00	2.38	0.00	4.76	0.79	0.00	7.94
	F3	0.00	0.00	0.00	0.00	0.00	0.00	0.00
Farm total		0.79	2.38	0.00	6.35	0.79	0.00	9.52
Overall total		1.59	24.60	7.94	20.63	23.02	5.56	81.75

**Figure 3:**
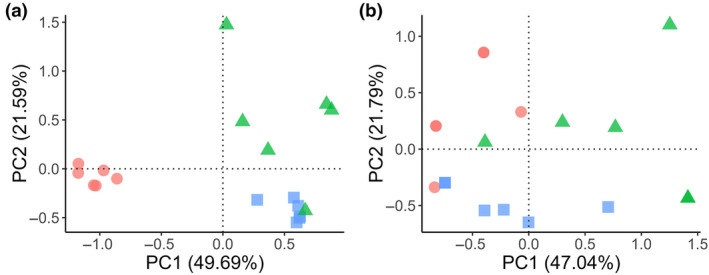
PCA of the principal components (PC) that explain the most variability between samples related to (a) haplotype composition and (b) relative haplotype presence by farm for the six most common haplotypes. Proportion of variability explained by each axis is presented in brackets within the axis label. Red circles = Dorset; Green triangles = Louth and Blue squares = Salisbury.

The abundance‐weighted haplotype composition was significantly different between farms (*P* < 0.0001), but was not significantly different between fields (*P* = 0.1214) nor sampling method (*P* = 0.7249). The PCA reveals visible grouping patterns based on farm origin, indicating that the haplotype compositions are most similar between samples of the same geographical origin. Samples from Salisbury and Louth farms show no differentiation across PC1, whereas Dorset displays significant dissimilarity [Fig. 3(a)]. The three farms occupied a separate portion along a continuum across PC2 with little overlap. Together, these axes show large interfarm and low interfield variability.

Results are similar for the relative richness with a significant farm effect (*P* < 0.0001), but no significant field effect (*P* = 0.1225) nor significant sampling method effect (*P* = 0.6120). In contrast to the haplotype composition PCA, the PCA for relative richness shows some grouping by farm, although with a much higher interfield variation [Fig. 3(b)].

The results suggest that both the relative haplotype presence and the composition of the haplotypes differ significantly between farms, but are not influenced by the sampling method used.

#### 3.3.2 Percentage of distinct haplotypes

Farm location significantly affected both the number of distinct haplotypes (*P* = 0.0307) and their proportion (*P* = 0.0207). Further analysis with Tukey's HSD test found that Dorset and Salisbury were not significantly differentiated from each other, whereas both were significantly differentiated from Louth (Table 4). The between‐field variability was not significant for either No. distinct (*P* = 0.5348) or *P*. distinct (*P* = 0.9492). Both sampling methods found the exact same number of distinct haplotypes and the percentage of distinct haplotypes did not significantly differ (*P* = 0.4175).

**Table 4:** Outcomes of a *post hoc* paired Tukey's honestly significant difference (HSD) test investigating the significant farm effect on the number and percentage of distinct haplotypes (No. distinct and P. distinct, respectively) found within commercial crops at three farms in the UK during April 2015

Comparison	Tukey's HSD adjusted *P*‐values	
	No. distinct	P. distinct
Louth–Dorset	0.0977	0.0343
Salisbury–Dorset	0.7131	0.9992
Salisbury–Louth	0.0302	0.0324

### 3.4 Additional survey findings

#### 3.4.1 CYP51 mutations

During this study a total of 31 distinct haplotypes were identified of which 10 have, to the best of our knowledge, not been reported previously (Table S2). Of the 33 published mutations in the CYP 51 gene for *Z. tritici*, 16 of these were found within this study together with three potential new mutations (see Table S3 for an overview of the percentage of isolates containing each of the mutations). Haplotypes contained between four and nine mutations.

#### 3.4.2 Cyp51 promotor alterations

PCR followed by sequencing was used to investigate alterations in the 5′ upstream regulation region. Only 3 (1.3%) isolates were shown to not contain an alteration to this region which is 334 bp in length. The majority of isolates (75.5%) recovered in this survey contained a larger insertion of ≈860 bp. Only in a few cases did isolates of the same haplotype have different sizes of insertion regions (Table S2).

## 4 DISCUSSION

This paper aimed to derive an appropriate sampling method to determine the population haplotype composition of the most common haplotypes, and to detect a greater number of rare haplotypes that could pose future resistance threats. The analysis took into consideration the between‐field and between‐farm variability and compared sampling using the conventional W sampling with high‐intensity sampling.

This study has discovered three potentially new mutations and 10 new haplotypes. Of the 33 mutations reported by Cools and Fraaije,^11^ this study found 16; five of which were previously reported as rare, and the other 11 as common or very common. The study showed that multiple haplotypes of *Z. tritici* are expected to be present at any given farm. In this study, 31 distinct *Z. tritici* haplotypes were observed across three commercial farms with ≤12 haplotypes observed in a single field. The haplotype population composition and richness of the most dominant haplotypes differed between farms but not between fields within a given farm. This suggests that the potential risk of yield loss owing to *Z. tritici* resistance could be assessed at the farm level without the need for a field‐specific assessment. However, assessments should not be based on national resistance data. None of the results were affected by whether sampling occurred using the conventional W method or point sampling at a single position within the field.

Intraseasonal spread of *Z. tritici* is believed to occur mainly through splash dispersal of asexual pycnidiospores leading to low per‐generation dispersal distances,^21^ a relatively high in‐field genotype diversity and relatively low interfield variation in genotype diversity.^22^ Our findings of a high number of distinct haplotypes found in each field (4–12 haplotypes per field) yet no difference in the frequency of distinct isolates of population composition of the most frequent haplotypes between fields, seem in agreement with this finding.

By contrast, the significant farm effects for all measures may have resulted from differences in environmental pressures as well as differences in fungicide product choice, leading to different selection pressures and hence the selection of divergent haplotype populations.

Given the findings of Ballu *et al*.^14^ that haplotype population composition is an important factor in the development of sustainable resistance management strategies this study highlights the role that the survey design for the detection of the haplotype composition has to play. Despite the clear need to determine haplotype composition and the detection of new resistance threats posed by novel mutations, some significant challenges remain hindering the design of practical farm‐based resistance management strategies. First, determining the population haplotype composition requires isolate sequencing which is currently still a very time‐intensive and expensive process. Secondly, a greater understanding of the link between haplotype and phenotype is required, given that haplotype frequency is not necessarily related to its level of fungicide sensitivity. For example, the review by Hellin *et al*.^13^ showed that the S524T mutation was very common across Europe and has been shown to be associated with a significant increase in resistance regardless of the other mutations present within the isolate. However, in this survey ≤72% of isolates sequenced at a single farm did not contain this mutation. Moreover, this mutation was not present in two of the six most common haplotypes. In these instances, other haplotypes may drive the field‐level resistance phenotype for which the resistance management and/or disease control strategies may need to be adjusted.

Unfortunately, there is currently a lack of systematic data to allow CYP51 haplotypes to be linked to azole resistance phenotypes.^23^ Additionally, most available data focuses on the phenotypic effects of individual mutations (see, for example, Hellin *et al*.^13^), which may miss potential synergistic and/or antagonistic effects. Given the large number of CYP51 mutations, generating such systematic data will be challenging and time‐consuming, but will be crucial in aiding farmers to apply the correct fungicide treatments to not only control *Z. tritici* in the short term, but also reduce resistance development in the long term. The work by Vagndorf *et al*.^24^ shows the potential for using different sources of varietal resistance in breeding programmes given their finding that some CYP51 mutations were significantly more frequent in isolates derived from certain varieties. Strategic fungicide resistance management using these types of approaches can have significant benefits, particularly in countries where azole resistance is still limited. In particular, when trying to manage selection in naïve populations or populations with frequencies of single resistance this approach could be complemented by a disease control strategy using a higher number of modes‐of‐action.^14^ Until these challenges have been addressed, a more suitable approach may be to screen for the presence of a single or low number of influential mutations using targeted multiplexed PCR assays^25^ or other in‐field diagnostic tools. The mutations included into such a screen could then be informed by national surveys screening for shifts in azole performance.^23^ Isolates found within this survey that have a reduced azole sensitivity then could be sequenced to check for novel mutations and haplotypes.

## Supporting information


**Table S1.** Overview of haplotypes found within commercial crops at three farms in the UK during April 2015. Results are presented for samples taken across three fields per farm and for two sampling methods used per field. H, point sampling (60 samples taken at a single position); W, conventional W‐shaped sampling (three samples taken at 20 positions across the W; 60 leaves in total).

**Table S2.** Haplotypes based on CYP51 mutations in *Zymoseptoria tritici* field populations identified during this study. The WT haplotype has been added for reference purposes and haplotypes highlighted in grey represent new findings.

**Table S3.** Mutation frequency of *Zymoseptoria tritici* in field populations on three commercial farms. Wheat plants were collected in April 2015 before treatment. Samples were collected from three fields on three UK commercial farms in Dorset, Salisbury (Wiltshire) and Louth (Lincolnshire), where STB control had failed the previous year.

## Data Availability

The data that support the findings of this study are available from the corresponding author upon reasonable request.
