# Supplementary material for: Appropriate sampling to aid on‐farm assessments of the haplotype composition of Zymoseptoria tritici populations
Source: Pest Manag Sci. 2024 Oct 11;81(2):599–606. doi: 10.1002/ps.8454 (PMC11716361; doi:10.1002/ps.8454)
Supplement: Supplementary file 1 — Table S1. Overview of haplotypes found within commercial crops at three farms in the UK during April 2015. Results are presented for samples taken across three fields per farm and for two sampling methods used per field. H, point sampling (60 samples taken at a single position); W, conventional W‐shaped sampling (three samples taken at 20 positions across the W; 60 leaves in total). [file PS-81-599-s003.docx]

**Table S1.** Overview of haplotypes found within commercial crops at three farms in the UK during April 2015. Results are presented for samples taken across three fields per farm and for two sampling methods used per field, H represents point sampling (60 samples taken at a single position) and W representing the conventional W-Shaped sampling (3 samples taken at 20 positions across the W; 60 leaves in total).
