# Supplementary material for: Appropriate sampling to aid on‐farm assessments of the haplotype composition of Zymoseptoria tritici populations
Source: Pest Manag Sci. 2024 Oct 11;81(2):599–606. doi: 10.1002/ps.8454 (PMC11716361; doi:10.1002/ps.8454)
Supplement: Supplementary file 2 — Table S2. Haplotypes based on CYP51 mutations in Zymoseptoria tritici field populations identified during this study. The WT haplotype has been added for reference purposes and haplotypes highlighted in grey represent new findings. [file PS-81-599-s001.docx]

**Table S2.** Haplotypes based on CYP51 mutations in *Z. tritici* field populations identified during this study. The WT haplotype has been added for reference purposes and haplotypes highlighted in grey represent new findings.

| **Haplo-type** | **50** | **134** | **136** | **188** | **284** | **379** | **381** | **410** | **459** | **460** | **461** | **513** | **524** | **165** | **377** | **454** | **Upstream regulation insert size** |
| --- | --- | --- | --- | --- | --- | --- | --- | --- | --- | --- | --- | --- | --- | --- | --- | --- | --- |
| WT | L | D | V | S | N | A | I | A | Y | G | Y | N | S | T | I | E | 0 |
| D13 |  |  | C |  |  |  | V |  |  |  | H |  | T |  |  |  | 800 |
| D26 | S |  |  |  |  |  | V |  |  |  | H |  |  |  | V |  | 800 |
| E3 | S |  | A |  |  |  | V |  |  |  | S |  | T |  |  |  | 800 |
| E4 | S | G | A |  |  |  | V |  |  |  | H |  |  |  |  |  | 800 |
| E5 | S |  | A |  |  |  | V |  |  |  | H |  | T |  |  |  | 800 |
| E8 | S |  | C | N |  |  | V |  |  |  | H |  |  |  |  |  | 800 |
| E9 | S | G | A |  |  |  | V |  | S |  |  |  |  |  |  |  | 0/800 |
| E25 |  |  | A |  |  | G | V |  |  |  | S |  | T |  |  |  | 0 |
| F2 | S |  |  | N |  |  | V |  | - | - |  | K |  |  |  |  | 120 |
| F4 | S |  | C | N |  |  | V |  |  |  | H |  | T |  |  |  | 800 |
| F6 | S |  | A | N |  |  |  |  | - | - |  |  | T |  |  |  | 800 |
| F7 | S |  | A |  |  | G | V |  |  |  | S |  | T |  |  |  | 0 |
| F8 | S | G | A |  |  |  | V |  |  |  | H |  | T |  |  |  | 800 |
| F17 | S | G | A |  |  |  | V |  |  |  | H |  |  |  |  | K | 800 |
| F19 | S |  | A | N |  |  | V |  |  |  | H |  | T |  |  |  | 800 |
| F20 | S |  | A |  |  |  | V |  | - | - |  | K |  |  |  |  | 120 |
| G1 | S |  |  | N |  | G | V |  | - | - |  | K |  |  |  |  | 800 |
| G2 | S |  |  | N |  | G | V |  | - | - |  |  | T |  |  |  | 800 |
| G3 | S |  | A | N |  |  |  |  | - | - |  | K | T |  |  |  | 800 |
| G9 | S |  | A | N |  |  | V |  | - | - |  | K |  |  |  |  | 120 |
| G10 | S |  | C | N |  |  | V |  | - | - |  |  | T |  |  |  | 800 |
| G11 | S |  | A | N |  |  | V |  | - | - |  |  | T |  |  |  | 800 |
| G12 | S |  | C | N |  |  | V |  | - | - |  | K |  |  |  |  | 120 |
| H3 | S |  |  | N | H | G | V |  | - | - |  | K |  |  |  |  | 0/800 |
| H4 | S |  | A | N |  | G | V |  | - | - |  |  | T |  |  |  | 800 |
| H5 | S |  |  | N |  | G | V | T | - | - |  | K |  |  |  |  | 800 |
| H6 | S |  | C | N |  | G | V |  | - | - |  |  | T |  |  |  | 800 |
| H10 | S |  |  | N |  | G | V |  | - | - |  |  | T | P |  |  | 800 |
| H11 | S |  | G | N |  | G | V |  | - | - |  |  | T |  |  |  | 800 |
| I1 | S |  | A | N |  | G | V |  | - | - |  | K | T |  |  |  | 800 |
| I4 | S |  | G | N |  | G | V |  | - | - |  | K | T |  |  |  | 0 |
