# Supplementary material for: Appropriate sampling to aid on‐farm assessments of the haplotype composition of Zymoseptoria tritici populations
Source: Pest Manag Sci. 2024 Oct 11;81(2):599–606. doi: 10.1002/ps.8454 (PMC11716361; doi:10.1002/ps.8454)
Supplement: Supplementary file 3 — Table S3. Mutation frequency of Zymoseptoria tritici in field populations on three commercial farms. Wheat plants were collected in April 2015 before treatment. Samples were collected from three fields on three UK commercial farms in Dorset, Salisbury (Wiltshire) and Louth (Lincolnshire), where STB control had failed the previous year. [file PS-81-599-s002.docx]

**Table S3.** Mutation frequency of *Z. tritici* in field populations on three commercial farms. Wheat plants were collected in April 2015 before treatment. Samples were collected from three fields on three UK commercial farms in Dorset, Salisbury (Wiltshire) and Louth (Lincolnshire), where STB control had failed the previous year.

| **Mutation** | **Percentage of isolates with mutation (%)** | **Observations (following information from Cools and Fraaije ^1^)** |
| --- | --- | --- |
| L50S | 97.4 | Very common, isolates without this mutation all had mutations at V136, Y461 and T524. |
| D134G | 27.9 | Always seen in combination with L50S, V136A, almost always with I381V and commonly in combination with S524T and a mutation at 461. |
| V136A | 63.9 | Always found with L50S and commonly with I381V (95.7%). |
| V136C | 9.9 | Always found in combination with I381V and commonly with S524T (98.3%) and L50S (89.7%). |
| V136G | 0.9 | Very rare mutation, found with L50S, S188N, A379G, I381V 459/460 deletion and S524T. |
| T165P | 0.4 | New mutation |
| S188N | 61.8 | Was always found in combination with L50S, and commonly in combination with I381V (95.3%) and Δ459/460 (83.7%). |
| N284H | 0.4 | Very rare mutation always found in combination with L50S, S188N, N248H, A379G, I381V, the 459/460 double deletion and N513K. |
| I377V | 0.9 | New mutation |
| A379G | 33.9 | Always found in combination with I381V and commonly with L50S (99.5%), S188N (97.2%) and Δ459/460 (97.2%). |
| I381V | 97.9 | Very common, isolates without this mutation all had mutations at L50S, V136A, S188N, 459/460 double deletion and S524T. |
| A410T | 0.4 | Rare mutation, always found with L50S, S188N, A379G, I381V, the 459/460 double deletion and N513K. |
| E454K | 0.4 | New mutation |
| Y459S | 0.4 | Very rare, was found in combination with L50S, D134G, V136A, and I381V. |
| ΔΔ459/ 460 | 59.7 | Found in combination with L50S (100%), S188N (98.6%), with I381V (97.2%). |
| Y461H | 39.1 | Always found in combination with I381V, commonly found with L50S 95.3%. |
| Y461S | 0.9 | Rare mutation always found in combination with L50S, V136A and S524T. |
| N513K | 27.0 | Always found with L50S and commonly found with the double deletion at 459/460 (99.3%), S188N (98.7%) and I381V (98.0%). |
| S524T | 48.5 | Common mutation, commonly seen with L50S (97.0%), V136A/C/G (98.4%) and I381V (94.8%). |

1. Cools HJ and Fraaije BA, Update on mechanisms of azole resistance in Mycosphaerella graminicola and implications for future control. *Pest Manag Sci*  **69**: 150-155 (2013).
